# Serum Procalcitonin (PCT) - Is there a Role as an Early Biomarker in Infected Diabetic Foot Ulcer (IDFU) Patients?

**DOI:** 10.5704/MOJ.2307.010

**Published:** 2023-07

**Authors:** J Omar, NS Ahmad, NAA Che-Soh, WN Wan-Azman, NM Yaacob, NS Abdul-Ghani, MR Abdullah

**Affiliations:** 1 Department of Chemical Pathology, Universiti Sains Malaysia, Kubang Kerian, Malaysia; 2 Department of Pathology, Hospital Bintulu Sarawak, Bintulu, Malaysia; 3 Department of Biostatistic, Universiti Sains Malaysia, Kubang Kerian, Malaysia; 4 Department of Orthopaedic, Universiti Sains Malaysia, Kubang Kerian, Malaysia; 5 Department of Community Medicine, Universiti Sains Malaysia, Kubang Kerian, Malaysia

**Keywords:** procalcitonin, biomarker, infected diabetic foot ulcer

## Abstract

**Introduction:**

Infected diabetic foot ulcers may lead to serious complications if not recognised in the early stage. Diagnosis of infection is particularly challenging at that stage; thus, a sensitive inflammatory biomarker may be helpful. We aimed to evaluate the role of procalcitonin (PCT) as an early biomarker for infected diabetic foot ulcers (IDFU).

**Materials and method:**

This cross-sectional study was conducted at Klinik Rawatan Keluarga (KRK), Orthopedic clinic and wards in Hospital Universiti Sains Malaysia (USM) from May 2020 to December 2020. A total of 264 participants were recruited and divided into three groups: 50 diabetic patients with no ulcers (control), 107 patients with non-infected diabetic foot ulcers (NIDFU), and 107 patients with infected diabetic foot ulcers (IDFU). The level of PCT was taken for all patients. Total white count (TWC) and C-reactive protein (CRP) were taken only for IDFU patients. Diagnosis of infection was based on the Infectious Disease Society of America-International Working Group of Diabetic Foot (IDSA-IMWGDF), and the severity of infection was graded according to the Wagner Classification.

**Results:**

The level of PCT was higher in IDFU than in NIDFU and diabetic patient, with a median (IQR) of 0.355 (0.63) ng/mL, 0.077 (0.15) ng/mL and 0.028 (0.02) ng/mL, respectively. PCT and CRP showed moderate positive correlations in IDFU patients (p<0.001). The sensitivity and specificity were 63.6% and 83.2%, respectively, at the best cut-off at 0.25 ng/mL.

**Conclusion:**

PCT is a valuable biomarker for the diagnosis of infection; however, it adds little value in the early diagnosis of IDFU in view of its low sensitivity.

## Introduction

Diabetic foot ulcer (DFU) is a major complication associated with diabetes mellitus and precedes amputation in up to 90% of cases^1^. Infection is the most frequent complication of DFU, and the diagnosis of infected diabetic foot ulcers (IDFU) is mainly based on clinical findings^2^. Because IDFU is progressive and associated with the potential risk of gangrene and limb amputation, prompt and accurate diagnosis is critical to reduce morbidity and mortality. More than 50% of all nontraumatic lower-limb amputations are due to diabetes, and the mortality following lower extremity amputation in diabetic patients ranges from 39% to 80% in five years^3^. The development of DFU and diabetic lower-limb amputation (DLLA) have multiple risk factors, including a previous history of foot ulcer, foot deformity, increasing age and duration of diabetes, treatment modality, alcohol intake, smoking, dyslipidemia, hypertension, and high body mass index (BMI). However, other studies have contradicted the association between these risks and the development of DFU and DLLA^4^.

Despite the recognition of the complications of delayed diagnosis and antibiotic commencement in IDFU, differentiating infected from uninfected ulcers remains challenging. This is because the severity and grading of the ulcer are often based only on clinical judgment, but the clinicians involved may differ in their experiences and exposures, thereby leading to divergence and contrasting discernment in diagnoses. In addition, the symptoms and signs of infection, such as pain, erythema, tenderness, and warmth, can be attenuated due to concomitant neuropathy and vasculopathy^5^. In contrast, conventional markers of infection and inflammation, such as a total white cell (TWC) and C-reactive protein (CRP), can be nonspecific. Given these limitations, identifying a reliable specific biomarker is warranted.

Currently, many studies are looking at procalcitonin (PCT) as a suitable and specific biomarker of bacterial infection to replace conventional markers. Procalcitonin (PCT) is a polypeptide consisting of 116 amino acids and is the prohormone of calcitonin. It is synthesised in thyroid C-cells or parafollicular cells, lungs, and pancreas. Generally, the level in the blood is very low or undetectable^6^. It is significantly elevated in bacterial infection but only slightly elevated by viral infection and non-infectious inflammatory diseases. It rises rapidly and peaks within 6 - 12 hours after an infectious insult and has systemic consequences^7^. PCT was described as a marker of sepsis in 1993^8,9^ and has been reported to be a superior marker for infection^10^. It has also been used as a prognostic marker for the outcome of infection^11^. Some randomised clinical trials have shown that PCT can guide antibiotic therapy in septic patients to provide a significant reduction in antibiotic administration^9,12^. PCT also has a role in assessing the severity of the disease caused by bacteria^13^. Recent studies have revealed that PCT could be a more potent marker for the diagnosis of bacterial infections in IDFU than conventional markers like TWC, CRP levels, and erythrocyte sedimentation rate (ESR)^14^.

Limited studies have used PCT to diagnose, treat, and monitor IDFU, while the results of these studies are varied and contradictory^15^. In this study, we evaluated the role of procalcitonin (PCT) as an early biomarker for diagnosis of infected diabetic foot ulcers (IDFU) and correlated PCT with conventional inflammatory markers (TWC and CRP) in the diagnosis of IDFU patients.

## Materials and Methods

This cross-sectional study as per STROBE guidelines16 was conducted between 1st of May 2020 and completed by 31st of December 2020 with ethical approval by the Human Research Ethics Committee of USM (JEPeM) (USM/JEPeM/19100636) in a tertiary teaching hospital. The control group consisted of 50 subjects with Type 2 Diabetes Mellitus (T2DM) regardless of duration were recruited from Klinik Rawatan Keluarga (KRK); all patients were at least 18 years old and met the World Health Organization (WHO) diagnostic criteria for T2DM^17^, and without any foot ulcers. The non-infected diabetic foot ulcer group (NIDFU) consisted of 107 subjects at least 18 years old with T2DM and NIDFU who came for scheduled appointments at the orthopaedic clinic. The infected diabetic foot ulcer group (IDFU) consisted of 107 subjects at least 18 years old with T2DM and IDFU who were admitted to the orthopaedic ward. IDFU diagnosis was based on the guideline of the Infectious Disease Society of America-International Working Group (IDSA-IWGDF) on Diabetic Foot Infection Classification^18^, which identifies two or more of the following signs: pain, warmth, tenderness, induration, and erythema or purulent secretion. The grade of ulcer severity was based on the Wagner Ulcer Classification^19^. Exclusion criteria were Type 1 Diabetes Mellitus or pregnancy-induced diabetes mellitus, concurrent systemic or localised infectious disease (e.g., urinary tract infection, pneumonia, or meningitis), presence of systemic inflammatory disease (e.g., inflammatory bowel disease, rheumatoid arthritis, or other rheumatic diseases), hematologic or solid organ malignancy, history of surgery ≤6 weeks before the date of recruitment, administration of antibiotic therapy ≤3 weeks prior to the date of recruitment and receiving immunosuppressive treatment^2,15^.

Venous blood was taken from the control and NIDFU groups during the patients’ scheduled appointments at the clinics. Venous blood was separated, and the plasma was kept in a freezer at -20°C until the samples were batch analysed for PCT in the Chemical Pathology laboratory. Leftover venous blood from IDFU patients in the wards was separated and the plasma was stored at -20°C freezer until batch analysis for PCT, TWC, and CRP in the Chemical Pathology, Haematology, and Immunology laboratories, respectively.

PCT levels were analysed using a Cobas e411 analyser (Roche Diagnostics; ELECSYS BRAHMS, Electrochemiluminescence Immunoassay Method, ECLIA) with a measurement range of 0.02–100ng/mL, a functional sensitivity of ≤0.06ng/mL (i.e., the lowest analyte concentration that can be reproducibly measured with an intermediate precision CV of 20%), a CV of 2.1%, and a lower limit of detection (LOD) ≤0.02ng/mL (i.e., the lowest measurable analyte level that can be distinguished from zero), as claimed by the manufacturer. CRP was analysed using QuickRead go (immunoturbidimetric method) with a measuring range of 5–200mg/L and a CV of 4.2%. TWC was run in the haematology laboratory using an automated cell analyser Sysmex XN1000 with a measuring range of 0.0– 440 × 103 and CV 3.0%.

Data analysis was performed using IBM SPSS software version 25. Categorical variables were reported as frequency (n) and percentage (%), while numerical variables were described as mean and standard deviation (SD) for the normally distributed analytes and median and interquartile (IQR) for not normally distributed analytes (checked using the Kolmogorov-Smirnov test of normality and a histogram with an overlaid normal curve). Categorical variable statistical differences between groups were assessed by the chi-square test. Pairwise comparisons of PCT between groups were analysed using the Kruskal Wallis test, and correlations between PCT and other inflammatory biomarkers were determined using the Spearman rho correlation coefficient. A receiver operating characteristic (ROC) curve was constructed, and the area under the ROC curve (AUC) was measured to evaluate the accuracy of PCT in discriminating IDFU from NIDFU. The Youden index (YI) was used to find the best cut-off for PCT sensitivity and specificity. A p-value of <0.05 was considered statistically significant.

## Results

A total of 264 patients were recruited for this study, including 50 diabetic patients without foot ulcers selected as the control group, 107 subjects classified as NIDFU, and 107 subjects classified as IDFU. The subjects ranged in age from 32 to 88 years, with a mean (SD) age of 60.70 (12.01), 57.94 (9.81), and 58.07 (11.45) for the control, NIDFU, and IDFU groups, respectively. The baseline characteristics of the study subjects are summarised in Table I. There was no significant difference between the three groups in terms of age (p=0.292) and sex (p=0.640).

**Table I:** Baseline characteristic of study subject

Variable	Group			Test stat (df)	P-value
	DM (n=50)	NIDFU (n=107)	IDFU (n=107)		
Age (years), mean (SD)	60.70 (12.01)	57.94 (9.81)	58.07 (11.45)	1.24 (2, 261)	0.292*
Sex					
Female	26 (52.0)	52 (48.6)	59 (55.1)	0.92 (2)	0.640†
Male	24 (48.0)	55 (51.4)	48 (44.9)		
Hypertension					
Yes	11 (22.0)	23 (21.5)	41 (38.3)	8.69 (2)	0.013†
No	39 (78.0)	84 (78.5)	66 (61.7)		
Chronic kidney disease					
Yes	42 (84.0)	76 (71.0)	89 (83.2)	5.80 (2)	0.058†
No	8 (16.0)	31 (29.0)	18 (16.8)		
HbA1c, median (IQR) %	6.3 (1.7)	9.0 (3.6)	8.3 (4.0)	28.74 (2)	<0.001‡
PCT, median (IQR), ng/mL	0.028 (0.02)	0.077 (0.15)	0.355 (0.63)	123.90 (2)	<0.001‡
CRP, mean (SD), mg/L	-	-	117.65 (69.55)	-	-
TWC, mean (SD), 10^9^	-	-	16.06 (7.47)	-	-

*One way analysis of variance; †χ2 test; ‡ Kruskal-Wallis

The wounds were characterised based on the location and grading of Wagner classification, there is a significant difference in grading and location of ulcer between NIDFU and IDFU as summarised in Table II, while the organisms isolated from wound cultures are summarised in Table III. There was a significant difference in term of ulcer location (p=0.008) and grading (p<0.001). The wound localisation for the majority of patients in NIFDU group was at the metatarsal area (42.1%), whereas for patients with IDFU, the most common area was at the toe area (39.2%).

**Table II:** Wound characteristics and grading of diabetic foot ulcer based on Wagner Ulcer Classification

Group	NIDFU (n=107), n (%)	IDFU (n=107), n (%)	χ2 statistics (df)	P-value
Grading				
1	85 (80.2)	8 (7.5)	139.8 (3)	<0.001
2	15 (14.2)	9 (8.4)		
3	1 (0.9)	55 (51.4)		
4	5 (4.7)	35 (32.7)		
Wound Localisation				
Toe	34 (31.8)	42 (39.2)	13.8 (4)	0.008
Metatarsal	45 (42.1)	22 (20.6)		
Midfoot/heel	15 (14.0)	18 (16.8)		
Toe-metatarsal	3 (2.8)	10 (9.4)		
Metatarsal-midfoot/heel	10 (9.3)	15 (14.0)		

* Data presented as column percentage

**Table III:** Organism isolated from wound culture of IDFU patients

Isolated organism	IDFU n=107 (%)
Not available	12 (11.2)
No growth	6 (5.6)
Mixed growth	13 (12.1)
Fungal	
Candida albican	1 (0.9)
Trichosporon asahii	1 (0.9)
Gram-positive bacteria	
Staphylococcus aureus	20 (18.7)
Coagulase-negative staphylococcus	1 (0.9)
Meticillin Resistant Staphylococcus aureus (MRSA)	1 (0.9)
Streptococcus beta-hemolytic:	
Group A	3 (2.8)
Group B	10 (9.3)
Group C	1 (0.9)
Group G	3 (2.8)
Streptococcus viridans	2 (1.9)
Streptococcus anginosus	1 (0.9)
Gram-positive cocci	2 (1.9)
Gram positive rod	3 (2.8)
Gram-negative bacteria	
Pseudomonas aeruginosa	10 (9.3)
Klebsiella pneumonia	4 (3.7)
Klebsiella pneumonia Extended Spectrum Beta	2 (1.9)
Lactamase (ESBL)	
Proteus mirabilis	2 (1.9)
Proteus mirabilis ESBL	1 (0.9)
Proteus hauseri	3 (2.8)
Burkholderia pseudomallei	1 (0.9)
E coli	1 (0.9)
Gram-negative anaerobe	3 (2.8)

**Fig 1:**
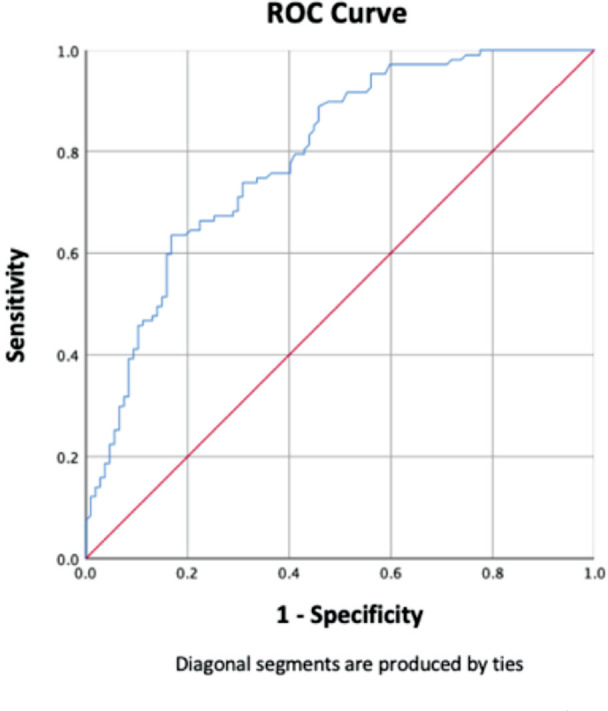
Receiving Operating Characteristic (ROC) curve for PCT for diagnosis of IDFU.

The baseline PCT level in control T2DM patients was 0.028 (0.02) ng/L. The PCT level was positively skewed to the right and was highest in IDFU patients, followed by NIDFU patients and then the control group, with median (IQR) values of 0.355 (0.63), 0.077 (0.15), and 0.028 (0.02), respectively. Pairwise comparisons of the PCT between the groups showed significant differences, as summarised in Table IV. All infective biomarkers were higher than the reference interval in the IDFU patients, with a mean (SD) of 16.06 (7.47) for TWC and 117.55 (69.55) for CRP, while the median (IQR) for PCT was 0.36 (0.63). PCT and CRP showed a moderate positive correlation (rs=0.506, P<0.001) but a low positive correlation with TWC (rs=0.353, P<0.001), as summarised in Table V^20^. The ROC curve analysis in Fig. 1 for predicting presence of infection demonstrated an AUC of 0.79 for PCT, a best cut-off at 0.25 ng/mL, and a sensitivity of 63.6%, specificity of 83.2%, positive predictive value (PPV) of 79.1%, negative predictive value (NPV) of 69.5%, positive likelihood ratio (LR+) 3.78, negative likelihood (LR-) 0.44, diagnostic odds ratio (DOR) 8.6 (95% CI, P-value <0.001).

**Table IV:** Comparison of PCT between the three groups

Pairwise comparison	Mean rank	Test statistic	P-value*
NIFDU vs. DM	117.3 vs. 46.5	5.41	<0.001
IDFU vs. DM	187.9 vs. 46.5	10.81	<0.001
IDFU vs. NIDFU	187.9 vs. 117.3	6.78	<0.001

*Mann Whitney test with Bonferroni correction for multiple comparisons. The overall Kruskal Wallis test P<0.001.

**Table V:** Correlation between PCT, CRP and TWC among patients with IDFU

Variables	Procalcitonin	Hs-CRP	TWC
Procalcitonin (ng/mL)	-	-	rs =0.353, P<0.001
CRP (mg/L)	rs=0.506, P<0.001	-	-
TWC (109)	rs =0.353, P<0.001	rs =0.548, P<0.001	-

rs = spearman correlation

## Discussion

Diabetic foot infection is the most common complication in diabetes mellitus. PCT has been considered a strong candidate as a biomarker of systemic bacterial infection and a strong acute inflammatory response that indicates deregulation even in patients that are not ill with sepsis. It has been considered a marker that could favour the diagnosis of IDFU together with other markers such as CRP, WBC and ESR.

PCT level in healthy individuals is low (<0.05–0.1ng/mL)^8,21^. However, its level is increased in the presence of inflammatory processes, such as DM, infection, autoimmune disease, and transplant rejection. Our study showed that the PCT level in our control group was within the level of a healthy population median (IQR) 0.028ng/ml, and this finding is in agreement with other studies, even though the demographic backgrounds differed. Researcher Umapathy *et al*^1^ and Jeandrot *et al*^22^ too, reported PCT levels of 0.04ng/mL in a similar control group; however, Korkmaz *et al*^15^ reported a slightly higher PCT level of 0.19ng/mL in their control group.

In our study, a statistically significant difference in PCT levels was observed among all the groups (the control, NIDFU, and IDFU groups), with a mean rank of 46.54, 117.31, and 187.86, respectively, (p-value <0.001), as shown in Table IV. This was due to the apparent ongoing inflammatory process of the ulcer. The highest level was seen in the IDFU group due to the presence of active infection. The PCT level in the NIDFU group was 0.077 (0.15) ng/mL, similar to the range reported in other studies^22-24^.

An elevated level of PCT in the IDFU group in our study, 0.355 (0.63) ng/mL which is near to the reported range of 0.2 - 0.27ng/mL from other studies by Jeandrot *et al*^22^, Uzun *et al*^23^, Zakariah *et al*^24^ and Van Asten *et al*^25^. Slightly high PCT levels in IDFU were found in studies by Umapathy *et al*^1^, Korkmaz *et al*^15^ and Reiner *et al*^26^, at 0.5ng/mL, 0.6ng/mL, and 0.7ng/mL, respectively. Massara *et al*^2^ reported the highest PCT level in IDFU, 2.92ng/mL, followed by Altay *et al*^27^ 1.4ng/mL and Jafari *et al*^28^ 1.2ng/mL. Hence, because of the different PCT levels reported in various studies, the use of PCT as a biomarker for localised bacterial infection was considered for DFU. A moderate positive correlation was noted between PCT and CRP in IDFU which was similar to other studies^6,24,27^.

Various cut-offs with variable sensitivity and specificity limit the use of PCT in IDFU. Uzun *et al*^23^ concluded that PCT had the greatest AUC, with a cut-off of 0.08ng/mL, a sensitivity of 77%, and a specificity of 100%. They reported that 22% of the patients with IDFU had below detectable functional sensitivity (<0.06ng/mL), suggesting that care must be taken when deciding not to use antibiotics. Jeandrot *et al*^22^ claimed that CRP was the most informative single parameter and that combining CRP with PCT provided the most relevant formula for distinguishing between NIDFU and IDFU ([0.162×CRP mg/L] + [17.437×PCT ng/mL]) with a cut-off of 4, a sensitivity of 90.9%, and a specificity of 82.6%. Jonaidi Jafari *et al*^28^ had 70% sensitivity and 74% specificity with the cut-off for PCT of 0.21ng/mL and in our study, for predicting the presence of infection demonstrated a sensitivity of 63.6%, specificity of 83.2%, at the best cut-off at 0.25ng/mL with AUC of 0.79.

Al-Shammaree *et al*^29^ claimed ESR was the best biomarker, followed by PCT, ANC, and WBC, with a PCT cut-off of 0.07ng/mL, a sensitivity of 87.5%, and specificity of 86.7%. Korkmaz *et al*^15^ found that CRP had the highest AUC, followed by fibrinogen, Il-6, ESR, and WBC, while PCT was ineffective in discriminating between IDFU and NIDFU. Umapathy *et al*^1^ found that PCT had the highest area AUC, with a cut-off ≥0.5ng/mL, a sensitivity of 54%, and specificity of 100%, followed by CRP, WBC, and ESR. Efat *et al*^5^ claimed that PCT was the best biomarker for the diagnosis of IDFU, with a sensitivity of 23.3% and specificity of 100%. Zakariah *et al*^24^ reported that hs-CRP had the highest AUC, followed by PCT and TWC, whereas the PCT cut-off was 0.11ng/mL, sensitivity was 70%, and specificity was 87%. Most researchers found CRP and ESR to be superior markers compared to PCT and hypothesised that CRP and ESR were the biomarkers of localised and mild infections^6,22,28,29^.

The role of PCT is more seen in identifying high-risk patients in improving clinician’s strategies such as the need for intensive care unit, reinforcement of antibiotics and close monitoring. It plays a significant role, especially in antibiotic stewardship, in reducing morbidity and mortality, length of stay in the hospital, and quality of life^30^, However, some studies have shown that PCT with low sensitivity (<80%), could cause patients with true infection who need antibiotics might be missed^1,15,24,28^.

This study had several limitations. One was that the diagnosis of infected versus non-infected DFU was solely based on clinical judgment. Consequently, interobserver variability could have occurred when diagnosing and grading the ulcers. The level of PCT also depends on the patient's age, time of assay, involved pathogen, and type of infection6 therefore, if the patient comes in early, the level of PCT might be lower than in other patients with the same severity who present for treatment much later. We suggest using IDSA-IWGDF Classification in future study to improve evaluation for grade of infection. Gram-negative bacterial infections also promote a higher level of PCT compared with gram-positive bacteria, this is due to lipoteichoic acid or lipopolysaccharide (LPS) that secreted by gram negative leads to production of TNF-α, IL-6 and IL-1β causing extensive transcription of calcitonin-mRNA and production of PCT^31^. As per Table I, there is a significant difference (p-value <0.05) between groups for presence of hypertension and level of HbA1c, perhaps can contribute as cofounder for difference in PCT level. This can hopefully be included in the future study to find confounder eg: gender, genetic variability, type of ulcer, duration of DM, body mass index (BMI) and other co-morbidities for level of PCT. We suggest taking serial PCT level to see the trend after initiation of antibiotic to assess usage of PCT for antibiotic stewardship in localised infection.

## Conclusion

In conclusion, PCT is a valuable biomarker. Levels of PCT were different between all groups, especially between NIDFU and IDFU. A positive correlation was seen between PCT with CRP and TWC in IDFU group. However, a wide range of PCT cut-off values with different sensitivity and specificity might not be the preferred choice in the diagnosis of IDFU. A low PCT sensitivity (<80%), could lead to misdiagnosis. The highest sensitivity would be to combine at least two biomarkers such as CRP and PCT or ESR and PCT to distinguish IDFU from NIDFU. On its own, PCT adds little value to the current practice and is not cost-effective in diagnosing IDFU.

## References

[1] Umapathy D, Dornadula S, Rajagopalan A, Murthy N, Mariappanadar V, Kesavan R (2018). Potential of circulatory procalcitonin as a biomarker reflecting inflammation among South Indian diabetic foot ulcers.. J Vasc Surg..

[2] Massara M, De Caridi G, Serra R, Barilla D, Cutrupi A, Volpe A (2017). The role of procalcitonin as a marker of diabetic foot ulcer infection.. Int Wound J..

[3] Atosona A, Larbie C (2019). Prevalence and Determinants of Diabetic Foot Ulcers and Lower Extremity Amputations in Three Selected Tertiary Hospitals in Ghana.. J Diabetes Res..

[4] Al Kafrawy NAEF, Mustafa EAAEA, Abd El-Salam Dawood AED, Ebaid OM, Zidane OMA. (2014). Study of risk factors of diabetic foot ulcers.. Menoufia Med J..

[5] Efat K, Morteza D, Allameh SF, Mehrnaz A, Manoochehr N, Alireza A (2018). Diagnostic Value of Serum Procalcitonin Level for Detecting Infected Diabetic Foot Ulcers.. Ann Med Health Sci..

[6] Park JH, Suh DH, Kim HJ, Lee YI, Kwak IH, Choi GW (2017). Role of procalcitonin in infected diabetic foot ulcer.. Diabetes Res Clin Pract..

[7] Greeff E (2012). Is procalcitonin useful in diagnosing septic arthritis and osteomyelitis in children?. SA Orthop J..

[8] Meisner M. (2014). Update on procalcitonin measurements.. Ann Lab Med..

[9] Khan F (2017). High Serum Procalcitonin: Interpret with Caution.. Clin Microbiol..

[10] Schuetz P, Christ-Crain M, Muller B (2009). Procalcitonin and other biomarkers to improve assessment and antibiotic stewardship in infections--hope for hype?. Swiss Med Wkly..

[11] Gonzalez-Busto Mugica I, Prieto Rodriguez J, Fernandez Fernandez A, Hueso Rieu R, Alvarez Menendez FV, Amigo Fernandez A (2011). Procalcitonin in the diagnosis of postoperative infection in knee arthroplasty.. Rev Esp Cir Ortop Traumatol..

[12] Samsudin I, Vasikaran SD (2017). Clinical Utility and Measurement of Procalcitonin.. Clin Biochem Rev..

[13] Velissaris D, Pantzaris ND, Platanaki C, Antonopoulou N, Gogos C (2018). Procalcitonin as a diagnostic and prognostic marker in diabetic foot infection. A current literature review.. Rom J Intern Med..

[14] Wacker C, Prkno A, Brunkhorst FM, Schlattmann P (2013). Procalcitonin as a diagnostic marker for sepsis: a systematic review and meta-analysis.. Lancet Infect Dis..

[15] Korkmaz P, Kocak H, Onbasi K, Bicici P, Ozmen A, Uyar C (2018). The Role of Serum Procalcitonin, Interleukin-6, and Fibrinogen Levels in Differential Diagnosis of Diabetic Foot Ulcer Infection.. J Diabetes Res..

[16] Vandenbroucke JP, von Elm E, Altman DG, Gotzsche PC, Mulrow CD, Pocock SJ (2014). Strengthening the Reporting of Observational Studies in Epidemiology (STROBE): explanation and elaboration.. Int J Surg..

[17] World Health Organization (WHO). (2020). HEARTS D: Diagnosis and management of type 2 diabetes.. https://www.who.int/publications/i/item/who-ucn-ncd-20.1.

[18] Lipsky BA, Berendt AR, Cornia PB, Pile JC, Peters EJ, Armstrong DG (2012). Infectious Diseases Society of America clinical practice guideline for the diagnosis and treatment of diabetic foot infections.. Clin Infect Dis..

[19] Mehraj DM (2018). A review of Wagner classification and current concepts in management of diabetic foot.. Int J Orthop Sci..

[20] Mukaka MM (2012). Statistics corner: A guide to appropriate use of correlation coefficient in medical research.. Malawi Med J..

[21] Rogic D, Juros GF, Petrik J, Vrancic AL (2017). Advances and Pitfalls in Using Laboratory Biomarkers for the Diagnosis and Management of Sepsis.. EJIFCC..

[22] Jeandrot A, Richard JL, Combescure C, Jourdan N, Finge S, Rodier M (2008). Serum procalcitonin and C-reactive protein concentrations to distinguish mildly infected from non-infected diabetic foot ulcers: a pilot study.. Diabetologia..

[23] Uzun G, Solmazgul E, Curuksulu H, Turhan V, Ardic N, Top C (2007). Procalcitonin as a diagnostic aid in diabetic foot infections.. Tohoku J Exp Med..

[24] Zakariah NA, Bajuri MY, Hassan R, Ismail Z, Md Mansor M, Othman H (2020). Is Procalcitonin more superior to hs-CRP in the diagnosis of infection in diabetic foot ulcer?. Malays J Pathol..

[25] Van Asten SA, Nichols A, La Fontaine J, Bhavan K, Peters EJ, Lavery LA (2017). The value of inflammatory markers to diagnose and monitor diabetic foot osteomyelitis.. Int Wound J..

[26] Reiner MM, Khoury WE, Canales MB, Chmielewski RA, Patel K, Razzante MC (2017). Procalcitonin as a Biomarker for Predicting Amputation Level in Lower Extremity Infections.. J Foot Ankle Surg..

[27] Altay FA, Sencan I, Senturk GC, Altay M, Guvenman S, Unverdi S (2012). Does treatment affect the levels of serum interleukin-6, interleukin-8 and procalcitonin in diabetic foot infection? A pilot study.. J Diabetes Complications..

[28] Jonaidi Jafari N, Safaee Firouzabadi M, Izadi M, Safaee Firouzabadi MS, Saburi A (2014). Can procalcitonin be an accurate diagnostic marker for the classification of diabetic foot ulcers?. Int J Endocrinol Metab..

[29] Al-Shammaree SAW, Abu-ALkaseem BA, Salman IN (2017). Procalcitonin levels and other biochemical parameters in patients with or without diabetic foot complications.. J Res Med Sci..

[30] Meloni M, Izzo V, Giurato L, Brocco E, Ferrannini M, Gandini R (2019). Procalcitonin Is a Prognostic Marker of Hospital Outcomes in Patients with Critical Limb Ischemia and Diabetic Foot Infection.. J Diabetes Res..

[31] Leli C, Ferranti M, Moretti A, Al Dhahab ZS, Cenci E, Mencacci A (2015). Procalcitonin levels in gram-positive, gram-negative, and fungal bloodstream infections.. Dis Markers..

